# PHYSIOLOGY OF SUCCESSFUL AGING: IMPLICATIONS FOR HEALTH AND WELL-BEING

**DOI:** 10.1093/geroni/igz038.1625

**Published:** 2019-11-08

**Authors:** Konstantinos Mantantzis, Denis Gerstorf, Thomas M Hess

**Affiliations:** 1 Humboldt University of Berlin, Berlin, Germany; 2 North Carolina State University, Raleigh, North Carolina, United States

## Abstract

Research into peripheral physiology and its association with cognition, emotionality, and social/physical functioning has received considerable attention over the years. However, many of the underlying mechanisms are not well understood. In this symposium, we have compiled a set of four empirical projects that showcase current and future endeavors to address some of the long-standing questions about when, how, and why physiology shapes and is shaped by key psychosocial resources. Hawkley et al. make use of data from the NSHAP and HRS longitudinal studies to investigate whether social relationships such as number of friends predicts risk of diabetes among older adults. Wilson et al. use dyadic data from young and middle-aged couples to examine cardiometabolic similarity among spouses, and how such concordance is shaped by key relationship factors such as emotional closeness. Pauly et al. use data from two daily-life studies of older couples to investigate how physiological synchrony in cortisol is modulated by partner interactions, empathy, and empathic accuracy. Finally, Mantantzis et al. make use of multi-year longitudinal data from the Berlin Aging Study II to examine the role of glucose regulation capacity for trajectories of subjective well-being among older adults. Thomas Hess will discuss the importance of these papers, discuss strengths and weaknesses of the approaches chosen, and consider implications for future research.

